# Optimal nitrogen regimes compensate for the impacts of seedlings subjected to waterlogging stress in summer maize

**DOI:** 10.1371/journal.pone.0206210

**Published:** 2018-10-23

**Authors:** Wenming Wu, Shiji Wang, Hongjian Chen, Youhong Song, Lin Zhang, Chen Peng, Lili Jing, Jincai Li

**Affiliations:** 1 Anhui Academy of Agricultural Sciences, Tobacco Research Institute/Maize Research Center, Hefei, P. R. China; 2 Anhui Agricultural University, School of Agronomy, Hefei, P. R. China; Fred Hutchinson Cancer Research Center, UNITED STATES

## Abstract

A field experiment was performed to explore the compensation effects of different nitrogen (N) regimes on the growth and photosynthetic capacity in different leaf layers of the summer maize hybrid of LuYu9105 under waterlogging at the seedling stage. The results showed that waterlogging significantly decreased the maximum green leaf area (gLA) by 10.0~15.3% and 9.3~22.5%, mainly due to the reduction in the below-ear layer leaves at the silking stage in 2014 and 2015, respectively. Waterlogging also significantly decreased the ear leaf photosynthetic rate (P_N_), and *F*_v_/*F*_m_, *F*_v_/*F*_o_, Φ_PSII_ and qP at the below-ear layer leaves at the mid- and late-filling stages, which was accompanied by a reduction in the duration of grain-filling (T) by 2.6~5.9%, thus resulting in a loss of grain yield by 7.0~18.5%. Interestingly, a shift in N from basal application to topdressing at the big flare stage was shown to compensate the adverse effects of waterlogging by through increased gLA and leaf photosynthetic capacity at the ear layer and the above-ear layer, as well as a greater grain-filling rate, resulting in an increase in grain yield by 9.9~27.0% and 17.8~25.8% compared to other N treatments. Therefore, this study showed that optimal nitrogen regimes during maize growth are capable of compensating for the impacts caused by waterlogging at the seedling stage.

## Introduction

Anhui Province is an important area for food supply in China, and maize is one of the major crops grown in the summer season. Summer maize is normally grown on mid-June after winter wheat has been harvested in a summer maize-winter wheat rotation area. Normally there is substantial rainfall from the mid-June that lasts nearly one month [1]. When the soil water content of the surface layer exceeds the field carrying capacity by at least 20%, this leads to free standing water on the soil surface [2, 3], and, consequently, maize seedling development is often subjected to waterlogging [1, 4].

Waterlogging has been shown to significantly affect maize growth and yield development [5–8], which depends on the genotype [9–11], the growth stage, and the duration of waterlogging [5, 7, 12]. Waterlogging inhibits the diffusion of gases through soil pores, and results in an enhancement of anaerobic respiration and accumulation of harmful substances in soil, which deteriorates rhizosphere environments [5, 13, 14], and restrains the growth and development of maize root, i.e., root length and number of root tips, causing a reduction of the absorption area [5, 15, 16]. Waterlogging also accelerates the leaf senescence process, via decreases in antioxidative enzyme activities and soluble protein content [7, 12, 17], resulting in the reduction of LAI, and disorders of leaf gas exchange and chlorophyll parameters [5, 18]. In addition, waterlogging restricts leaf photosystem II (PSII) photosynthetic activity and blocks photosynthetic electron transport [19], which reduces the quantum efficiency of PSII actual electron transfer and the photosynthetic rate (P_N_) [5, 20]. Previous study show that the third leaf stage of maize is most susceptible to waterlogging [5]. The adverse effects of waterlogging in the early stage have much longer-term effects, and these adverse effects are still observed during the later growth period, resulting in a decrease in the aboveground matter accumulation, nutrient absorption and transition, grain filling rate, and grain yield [5, 19, 21–23]. However, previous studies have mainly focused on whole canopy response to waterlogging, and little is known about how different canopy layers responded. Leaves of maize plant can be classified into three groups: above-ear, ear and below-ear layers, which have different functions. How waterlogging impacts the different leaf layers still need further research.

Tackling waterlogging mainly depends on the following aspects: (1) to choose the water-resistant hybrids [9, 10, 24, 25]; (2) to investigate the compensate effects of biochemical preparation on the growth and production [17, 26]; (3) to explore new planting management to overcome the waterlogging disaster, such as ridge tillage or different drainage measurement [6, 27]. Previous researches also suggest to determine the waterlogging risk in fields with underlying impermeable layers that enhance lateral flow of water [28]. Nevertheless, how to change the fertilization method to alleviate the adverse effects of waterlogging on growth and grain yield of summer maize remains to be determined. N metabolism has been shown to contribute to cellular acclimation to low oxygen stress in plants [29]. In waterlogged soil, N losses from the soil [30], a lack of N and the decline of root absorption ability can cause a reduction in the leaf chlorophyll content and accelerate leaf senescence [31]. In contrast, an increase in N availability may improve photosynthetic capacity or stomatal control under water and N deficit conditions [32–35]. Furthermore, it has also been reported that N supply can increase the effective quantum yield of photochemical energy conservation in PSII [5, 36]. As a consequence, this is suggestive of compensating maize leaf functioning after waterlogging using N regimes. In the Anhui province, N fertilization is mainly 100% basal fertilizer. Due to the adverse effects of waterlogging, it can be seen that a shift of N from basal application to topdressing at the flare stage may compensate for waterlogging for the growth of summer maize. In this context, we conducted a 2-year field experiment to assess the adverse effects of waterlogging on the growth and development of different leaf layers, and the compensating effects of N regimes. Accordingly, the objectives of this study were i) to explore the adverse effects of waterlogging on the gLA, photosynthetic capacity and chlorophyll characteristics of different leaf layers; ii) to examine how different N regimes compensated for the adverse effects of waterlogging on maize growth and development.

## Materials and methods

### Plant materials and culture conditions

A field trial was conducted at the experimental station of the Anhui Academy of Agricultural Sciences, China (31°57′N, 117°11′E), in 2014 and 2015, using the summer maize hybrid “LuYu 9105”, which is one of the most widely grown hybrids in Anhui Province. The region has a temperate continental monsoon climate. The topsoil (0–20 cm) is yellow cinnamon soil, containing 21.6 g kg^−1^ organic matter, 118.4 mg kg^−1^ alkali-hydrolyzable N, 25.4 mg kg^−1^ available P, and 269.6 mg kg^−1^ available K.

### Experimental design

In this experiment, a randomized complete block design was used to arrange two water supply treatments, i.e., waterlogging stress and no waterlogging (the control), with four replicates. Waterlogging was applied for 7 days from the fourth leaf stage. In the waterlogged treatment, 1 cm of water was maintained above the soil surface using a water valve to control water flow throughout the waterlogging period. Each plot was 3.6 m×6.7 m. There was a 3.0 m interval between two adjacent experimental plots and a ridge with a height of 0.4 m along the border of each plot. An independent water supply pipe was provided in each waterlogging plot to achieve waterlogging during the seedling stage; in contrast, the control treatment was rainfed and was not irrigated during this stage. The planting density was 75,000 plants ha^-1^. Four N regimes (N1, N2, N3 and N4) were designed for the waterlogging and control treatments. The N fertilizer was urea, and the application rate was 240 kg ha^-1^ for all treatments but with different proportions at the basal, jointing, and big flare stages (10:0:0 for N1, 7:3:0 for N2, 5:5:0 for N3, and 3:5:2 for N4). Fertilizers containing phosphorus (P_2_O_5_) and potassium (K_2_O) were applied at respective rates of 105 kg ha^-1^ and 135 kg ha^-1^ per plot prior to sowing. After the waterlogging treatment was applied for 7 days, the water in the plot was drained, afterwards, the management of the waterlogging treatment was the same as in the control treatment. Weeds, diseases, and insect pests were rigorously controlled.

### Green leaf area (gLA)

Leaves were grouped into three leaf layers, i.e., ear layer leaves (three-ear leaves), above-ear layer leaves (the leaves above the three-ear leaves), and below-ear layer leaves (the leaves below the three-ear leaves). At the silking stage, four plants were randomly selected in each plot to determine leaf area. gLA per plant = ∑A×B×0.75.

A is the leaf length, B is the maximum leaf widt.

### Leaf N concentration

Fresh samples were heated in an oven for 30 min at 105°C to deactivate enzymes and were then dried at 70°Cto a constant weight. The dried samples were ground to determine the N concentration. Plant samples were digested using the H_2_SO_4_–H_2_O_2_ method, and the total N concentration was measured using a continuous flow auto-analyzer (AAIII; SEAL Analytical, Germany).

### Leaf gas exchange characteristics

At the mid- and late-filling stages, the net photosynthetic rate (P_N_), stomatal conductance (g_s_), transpiration rate (T_r_), and intercellular CO_2_ concentration (c_i_) of the ear leaves were measured using a portable gas exchange system (LI-6400, LI-COR, Lincoln, USA). Three plants from each plot were measured on clear days between 9:30–11:00 A.M. Measurement conditions remained consistent: LED light source, PAR of 1400 μmol m^−2^, flow rate of 500 cm^3^ min^–1^, constant CO_2_ concentration of 360 μmol mol^−1^, air temperature of 30°C, and relative humidity of 65%.

Chlorophyll fluorescence was determined on above-ear leaves, ear leaves and below-ear leaves by non-destructively measuring photo-physiological parameters using pulse amplitude modulation fluorometry (PAM-2500, Walz, Germany) at the silking stage, mid-filling stage and late-filling stage. Initial fluorescence (*F*_0_) and maximal fluorescence (*F*_m_) were measured after 30 min of dark adaptation. The intensity of the saturation pulses used to determine the maximal fluorescence emission in the presence (*F*_m_’) and absence (*F*_m_) of quenching was 4000 μmol (photon) m^–2^ s^–1^ for 0.8 s, whereas “actinic light” was 600 μmol (photon) m^–2^ s^–1^. Steady-state fluorescence (*F*_s_), basic fluorescence after light induction (*F*_0_’), maximal PSII photochemical efficiency (*F*_v_/*F*_m_), effective quantum yield of PSII (Ф_PSII_), and photochemical (qP) fluorescence quenching coefficients were also recorded.

### Grain filling characteristics

The silking date was recorded when the silks emerged in 50% of the plants in a plot. Three ears of every plot from randomly selected plants were harvested at 10, 20, 30, 40, 50 and 60 days after anthesis and 100 grains were cut from the middle of each sampled ear [37]. The dry weight of 100 grains for each ear was measured after drying to a constant weight in a forced air oven at 80°C.

The dynamics of grain weight during grain filling followed Richards' growth equation [38]:

$$Y=\frac{K}{1+e^{A+Bt}}$$

where Y is the grain weight, K is the ultimate grain weight, t is the day after pollination, and A and B are coefficients determined by regression.

The data of the early grain filling period was $t_{1}=\frac{A-\ln(2+1.732)}{\left(-B\right)}$, the middle grain filling period was $t_{2}=\frac{A+\ln(2+1.732)}{\left(-B\right)}$, the late grain filling period was $t_{3}=\frac{-(4.59512+A)}{B}$, the duration of the early grain filling period was T_1_ = t_1_, the duration of the middle grain filling period was T_2_ = t_2_−t_1_, the duration the late grain filling period was T_3_ = t_3_−t_2_, the grain-filling duration was T = t_3_, the mean grain-filling rate was $V_{a}=\frac{K}{t_{3}}$, the maximum grain-filling rate was $V_{m}=\frac{\left(-B\times K\right)}{4}$, the grain weight during the early grain filling period was $W_{1}=\frac{K}{\left(1{+e}^{A+Bt_{1}}\right)}$, the grain weight during the middle grain filling period was $W_{2}=\frac{K}{1+e^{A+Bt_{2}}}-\frac{K}{1+e^{A+Bt_{1}}}$, the grain weight during the late grain filling period was $W_{3}=\frac{K}{1+e^{A+Bt_{3}}}-\frac{K}{1+e^{A+Bt_{2}}}$, the grain-filling rate during the the early grain filling period was $V_{1}=\frac{W_{1}}{T_{1}}$, the grain filling rate during the middle grain filling period was $V_{2}=\frac{W_{2}}{T_{2}}$, the grain filling rate during the late grain filling period was $V_{3}=\frac{W_{3}}{T_{3}}$.

### Grain yield

At the maturity stage, 30 cobs were harvested from three rows at the center of each plot to determine the yield and ear characteristics, including ear length, row number, and kernel number per row. All kernels were air-dried, and grain yield was calculated at 14% moisture, which is the standard for maize storage or sale in China (GB/T29890-2013). Grain yield (kg ha^−1^) = harvested ears (ears ha^−1^) × kernel number per ear × 1000-grain weight (g 1000 grains^−1^)/10^6^ × (1– sample moisture content %) / (1–14%).

### Statistical analysis

The effects of waterlogging stress, N treatments, and their interactions were analyzed using two-way analysis of variance (ANOVA; *p* = 0.05). Differences between the means of the waterlogging and N treatments were compared using LSD multiple-range tests at 0.05 probability levels. Statistical analyses were performed using SPSS 13.0 (SPSS, Chicago, IL, USA), and all figures were drawn using SigmaPlot 10.0.

## Results

### gLA

**Fig 1:**
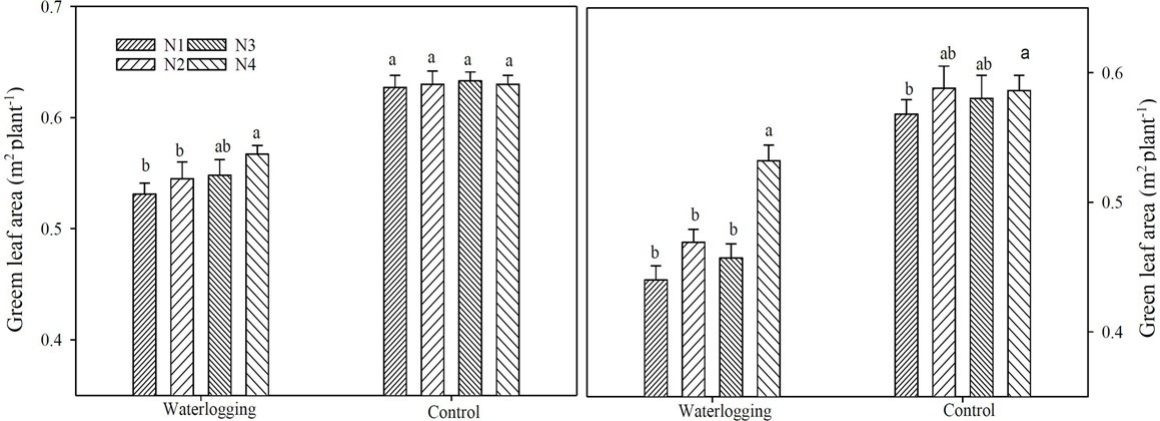
Green leaf area at the silking stage across differing nitrogen regimes. The error bars represent the standard errors of the mean. Different letters above the bars indicate significant differences (p < 0.05) among different N regimes. N1: 100% N applied as basal fertilizer, N2: 70% N applied as basal fertilizer and 30% N applied at the jointing stage, N3: 50% N applied as basal fertilizer and 50% N applied at the jointing stage, N4: 30% N applied as basal fertilizer, 50% N applied at the jointing stage and 20% N applied at the big flare stage.

**Fig 2:**
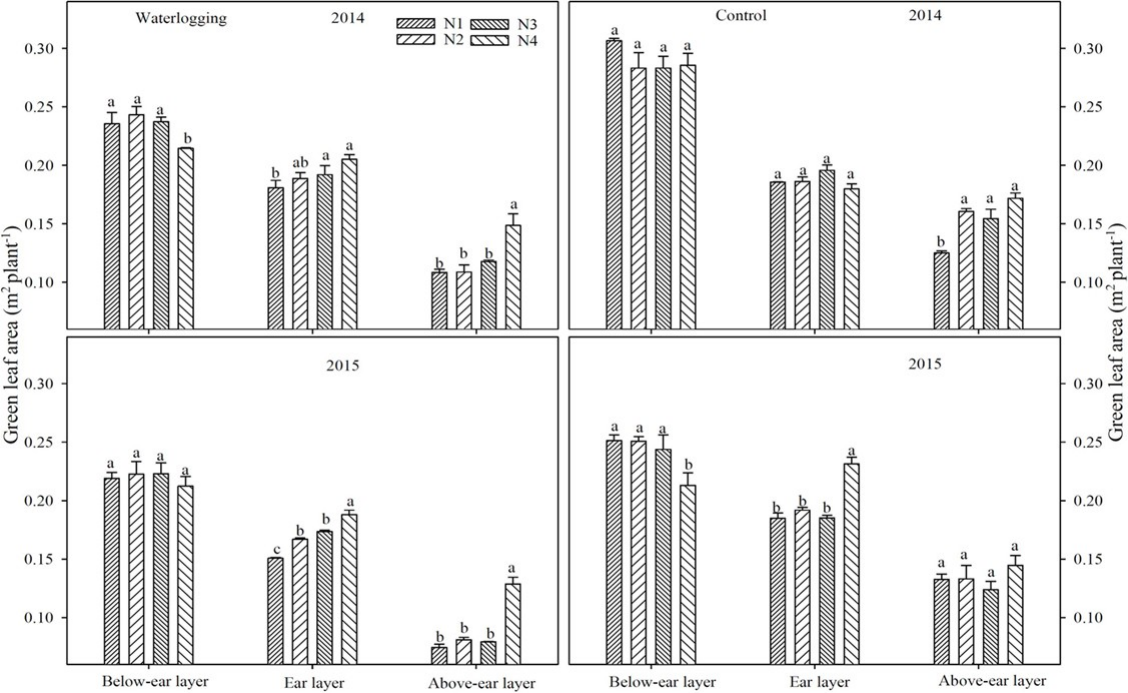
Green leaf area of different leaf layers at the silking stage. The error bars represent the standard errors of the mean. Different letters above the bars indicate significant differences (p < 0.05) among different N regimes. N1: 100% N applied as basal fertilizer, N2: 70% N applied as basal fertilizer and 30% N applied at the jointing stage, N3: 50% N applied as basal fertilizer and 50% N applied at the jointing stage, N4: 30% N applied as basal fertilizer, 50% N applied at the jointing stage and 20% N applied at the big flare stage.

Waterlogging significantly decreased gLA by 14.5, 14.3, 13.2 and 9.9% in 2014, and 22.6, 20.7, 14.9 and 9.7% in 2015 in different N treatments, respectively, compared to the corresponding control (Fig 1). Waterlogging decreased gLA by 14.1~24.9% and 0.3~12.9% in the below-ear layer, by 1.9~2.6% and 6.3~18.7% in the ear layer, by 13.4~32.2% and 11.0~43.8% in the above-ear layer, compared to the corresponding controls in 2014 and 2015, respectively (Fig 2). N shifted from basal application to topdressing at the big flare stage effectively improved gLA under waterlogging. The gLA of the N4 treatment was significantly higher than that of the other N treatments in the ear layer by 6.8~24.8% and in the above-ear layer by 26.2~72.7% under waterlogging, 7.0~37.4% in the above-ear layer of the control in 2014 and 2015, respectively.

### Leaf nitrogen concentrations

Waterlogging resulted in a significant reduction in N concentration in the different leaf layer leaves (Table 1). Specifically, waterlogging reduced the N concentration by 5.1 and 22.1% in the below-ear layer, 2.9 and 12.1% in the ear layer, and 6.7 and 15.3% in the above-ear layer leaves at the silking stage and by 14.4, 9.5, and 6.2% and 14.0 3.2, and 12.8% of the below-ear layer, the ear layer and the above-ear layer leaves at the maturity stage in 2014 and 2015, respectively. Waterlogging decreased N concentrations in below-ear layer leaves by the greatest amount. However, in three of the regimes (N2, N3 and N4), where N shifted from a basal application to topdressing, the effect of waterlogging on the N concentration was effectively alleviated. Among these regimes, the N4 treatment at the respective silking and maturity stages had the greatest effect, and the values were increased by 23.5, 21.8, and 9.0% and 8.5, 36.7, and 25.4% at the silking stage, and by 41.6, 47.1, and13.6% and 48.4, 7.9, and 33.4% at the maturity stage in the below-ear layer, the ear layer and the above-ear layer, compared to the N1 treatment under waterlogging in 2014 and 2015, respectively.

**Table 1:** Leaf N concentrations in different leaf layers at the silking and maturity stages.

Treatment		2014			2015		
		Below-ear layer	Ear layer	Above-ear layer	Below-ear layer	Ear layer	Above-ear layer
Waterlogging							
Silking stage	N1	2.03±0.07 b	2.15±0.13 b	2.52±0.05 b	1.84±0.04 b	2.00±0.04 c	2.10±0.03 c
	N2	1.94±0.07 b	2.48±0.11 a	2.64±0.03 ab	1.78±0.04 b	2.39±0.11 b	2.30±0.04 b
	N3	2.34±0.07 a	2.50±0.07 a	2.68±0.04 ab	1.85±0.02 b	2.41±0.10 b	2.55±0.03 a
	N4	2.50±0.08 a	2.62±0.06 a	2.75±0.07 a	2.00±0.02 a	2.74±0.06 a	2.63±0.07 a
Maturity stage	N1	0.73±0.04 c	1.13±0.05 c	1.38±0.06 b	0.87±0.04 b	1.45±0.09 a	1.05±0.09 c
	N2	1.24±0.04 a	1.47±0.03 b	1.31±0.02 b	0.93±0.01 b	1.30±0.11 a	1.56±0.10 a
	N3	1.06±0.10 ab	1.34±0.10 b	1.39±0.07 b	0.99±0.09 b	1.42±0.20 a	1.25±0.09 b
	N4	1.03±0.05 b	1.67±0.09 a	1.56±0.04 a	1.29±0.04 a	1.56±0.12 a	1.41±0.11 ab
Control							
Silking stage	N1	2.30±0.04 a	2.52±0.19 a	2.85±0.07 a	2.55±0.11 a	2.69±0.14 a	2.80±0.11 a
	N2	2.01±0.11 b	2.45±0.09 a	2.57±0.03 b	2.39±0.04 a	2.62±0.19 a	2.84±0.15 a
	N3	2.46±0.15 a	2.51±0.06 a	2.96±0.15 a	2.43±0.06 a	2.74±0.28 a	2.78±0.11 a
	N4	2.51±0.09 a	2.56±0.13 a	2.98±0.10 a	2.21±0.19 a	2.80±0.01 a	2.90±0.19 a
Maturity stage	N1	1.13±0.03 b	1.43±0.05 b	1.30±0.03 c	1.17±0.10 b	1.25±0.05 c	1.21±0.09 c
	N2	1.01±0.04 b	1.5±0.12 ab	1.45±0.04 b	1.09±0.06 b	1.52±0.13 b	1.60±0.10 ab
	N3	1.32±0.03 a	1.52±0.10 ab	1.48±0.05 b	1.09±0.05 b	1.34±0.04 bc	1.41±0.06 bc
	N4	1.29±0.08 a	1.75±0.08 a	1.79±0.04 a	1.39±0.05 a	1.81±0.07 a	1.83±0.06 a

Values followed by different letters are significantly different (*p* < 0.05) among different N regimes. N1: 100% N applied as basal fertilizer, N2: 70% N applied as basal fertilizer and 30% N applied at the jointing stage, N3: 50% N applied as basal fertilizer and 50% N applied at the jointing stage, N4: 30% N applied as basal fertilizer, 50% N applied at the jointing stage and 20% N applied at the big flare stage.

### Leaf photosynthetic capacity

Waterlogging led to a noticeable decrease in P_N_, g_s_ and T_r_ at the mid-filling stage (16.1, 25.0, and 5.1%) and at the late-filling stage (28.9, 12.1, and 10.9%, respectively) compared to the corresponding control. An increase in c_i_ was observed in maize subjected to waterlogging, with 19.3% greater values at the mid-filling stage and 61.3% greater values at the late-filling stage. Compared to the mid-filling stage, P_N_, g_s_ and T_r_ decreased by 38.9, 31.1 and 21.8% in the late-filling stage under waterlogging, whereas in the control, P_N_, g_s_ and T_r_ decreased by approximately 27.9, 41.2 and 16.8%, respectively (Table 2).

**Table 2:** Effects of waterlogging imposed at the seedling stage on photosynthetic capacity at the grain-filling stage (2015).

	Mid-filling stage			Late-filling stage		
	Waterlogging	Control	Amplitude	Waterlogging	Control	Amplitude
Net photosynthetic rate (P_N_) μmol m^-2^s^-1^	22.1±1.2 b	26.3±0.5 a	16.1↓	13.5±1.6 b	19.0±2.3 a	28.9↓
Stomatal conductance (g_s_) mmol m^-2^s^-1^	157.8±4.5 b	210.3±13.3 a	25.0↓	108.7±8.1 b	123.7±9.7 a	12.1↓
Intercellular CO_2_ concentration (c_i_) μmol mol^-1^	101.8±26.3a	85.3±9.7 b	19.3↑	127.8±18.2 a	79.2±20.1 b	61.3↑
Transpiration rate (T_r_) mmol m^-2^s^-1^	3.6±0.2 a	3.8±0.1 a	5.1 ↓	2.8±0.2 a	3.2±0.2 a	10.9↓

Values followed by different letters are significantly different (*p* < 0.05) between the waterlogging treatment and the control.

At the mid-filling stage, there was no significant difference among different N regimes for P_N_, g_s_ and T_r_. At the late-filling stage, these characteristics showed higher values in N4 than in N1 and N2, with increases of 63.3 and 55.3% (P_N_), 43.2 and 7.0% (g_s_), and 34.5 and 12.3% (T_r_) compared to N1 and N2 under waterlogging, and 80.6 and 8.0% (P_N_), 47.7 and 19.6% (g_s_), and 25.0 and 2.9% (T_r_) compared to the control, respectively (Table 3).

**Table 3:** Effects of different N regimes on the photosynthetic capacity of summer maize subjected to waterlogging stress at the seedling stage (2015).

Treatment		Net photosynthetic rate (P_N_) μmol m^-2^s^-1^		Stomatal conductance (g_s_) mmol m^-2^s^-1^		Intercellular CO_2_ concentration (c_i_) μmol mol^-1^		Transpiration rate (T_r_) mmol m^-2^s^-1^	
		Waterlogging	Control	Waterlogging	Control	Waterlogging	Control	Waterlogging	Control
Mid-filling stage	N1	20.2±1.9 a	25.8±2.3 a	148.0 ± 30.0 a	180.4±3.8 b	180.7±10.8 a	104.8±25.9 a	3.5±0.2 a	3.9±0.2 a
	N2	23.3±2.6 a	26.5±4.2 a	153.0±33.4 a	195.6±32.1 ab	79.4±26.1 b	60.6±3.3 b	3.2±0.5 a	3.8±0.7 a
	N3	19.9±3.4 a	25.3±0.6 a	162.2±29.2 a	229.3±70.0 ab	72.6±9.7 b	79.8±13.0 ab	3.9±0.5 a	3.6±0.4 a
	N4	24.9±2.0 a	27.7±1.0 a	167.9±29.8 a	236.1±6.3 a	74.4±19.4 b	84.4±11.5 a	4.0±0.2 a	4.0±0.5 a
Late-filling stage	N1	11.0±2.0 b	12.4±2.4 b	85.3±14.5 b	98.7±13.5 b	155.4±12.0 a	138.9±19.3 a	2.4±0.3 b	2.8±0.3 b
	N2	11.6±2.9 b	20.6±0.8 a	114.1±20.8 a	121.9±11.9 a	157.1±4.4 a	53.8±19.9 b	2.9±0.4 a	3.4±0.3 a
	N3	13.4±1.5 ab	20.5±2.7 a	113.2±3.0 a	128.2±38.5 a	118.5±8.0 b	56.7±37.1 b	2.8±0.3 ab	3.1±0.6 ab
	N4	18.0±0.2 a	22.4±2.9 a	122.1±14.8 a	145.8±15.0 a	80.2±26.0 c	67.4±4.4 b	3.3±0.4 a	3.5±0.2 a

Values followed by different letters are significantly different (*p* < 0.05) among different N regimes.N1: 100% N applied as basal fertilizer, N2: 70% N applied as basal fertilizer and 30% N applied at the jointing stage, N3: 50% N applied as basal fertilizer and 50% N applied at the jointing stage, N4: 30% N applied as basal fertilizer, 50% N applied at the jointing stage and 20% N applied at the big flare stage.

### PSII photochemical characteristics

**Fig 3:**
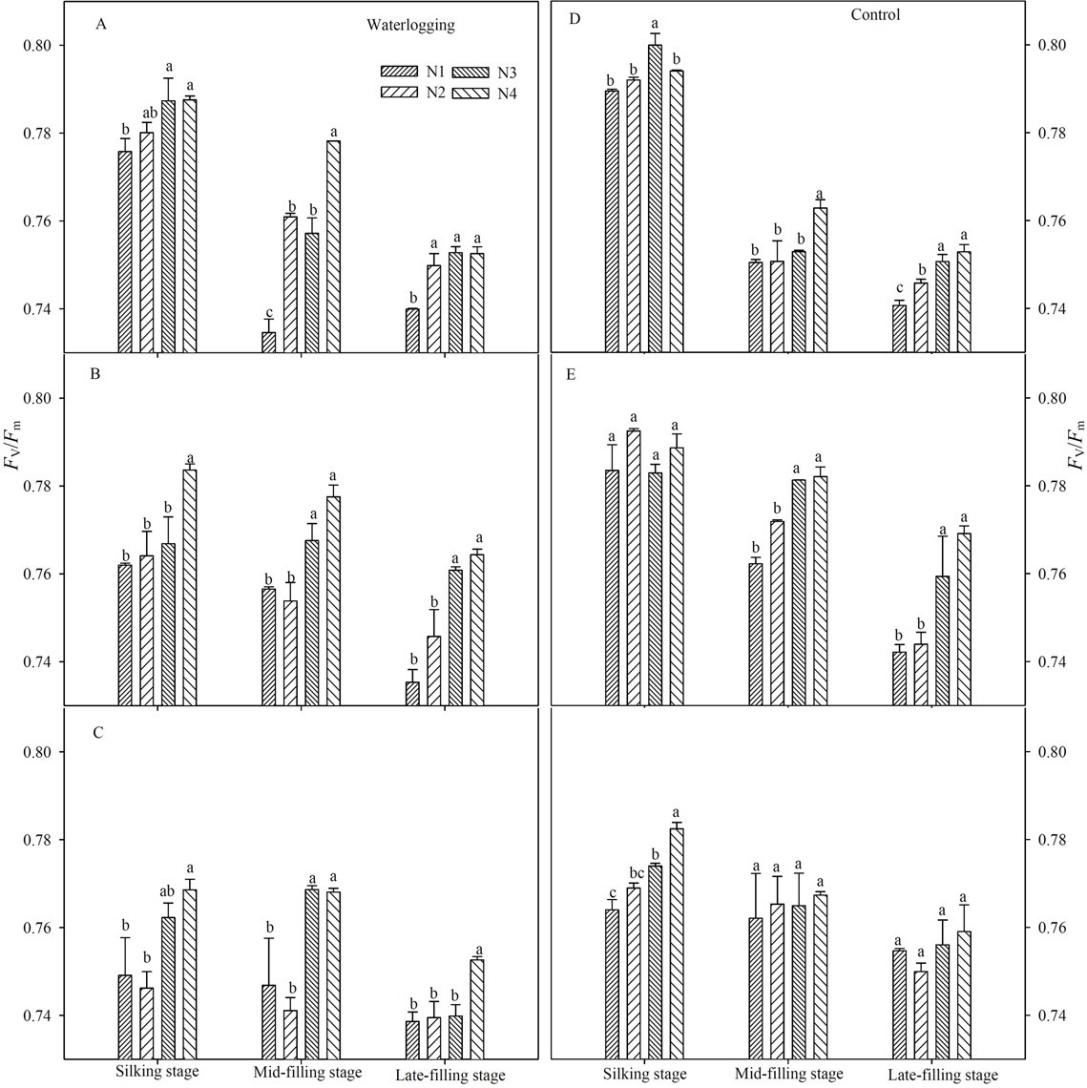
Maximal efficiency of PSII (*F*_v_/*F*_m_) in different leaf layers under different N treatments (2014). The error bars represent the standard errors of the mean. Different letters above the bars indicate significant differences (*p* < 0.05) among the different N treatments. A,D: above-ear leaves; B,E: ear leaves; C,F: below-ear leaves. N1: 100% N applied as basal fertilizer, N2: 70% N applied as basal fertilizer and 30% N applied at the jointing stage, N3: 50% N applied as basal fertilizer and 50% N applied at the jointing stage, N4: 30% N applied as basal fertilizer, 50% N applied at the jointing stage and 20% N applied at the big flare stage.

**Fig 4:**
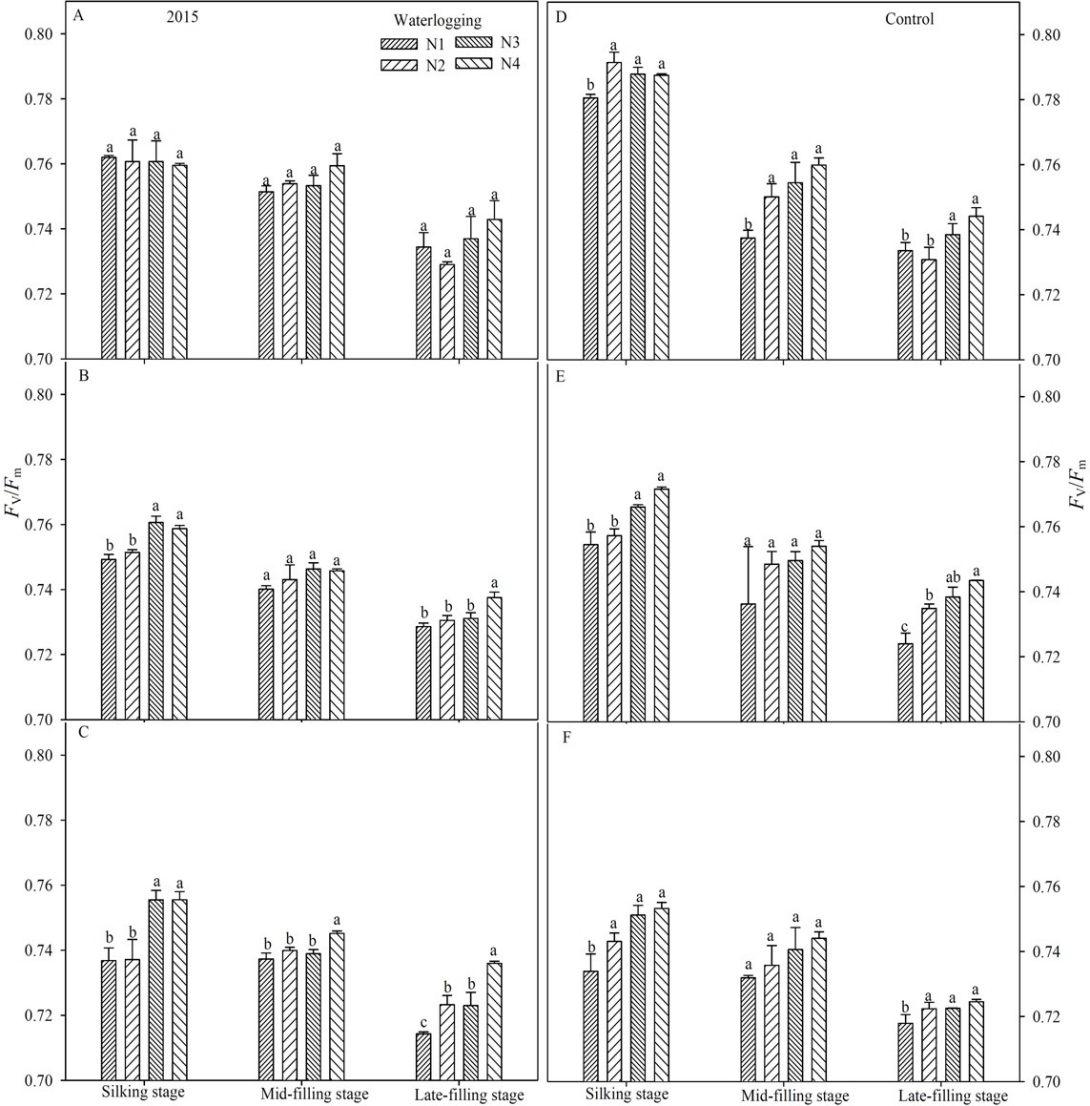
Maximal efficiency of PSII (*F*_v_/*F*_m_) in different leaf layers under different N treatments (2015). The error bars represent the standard errors of the mean. Different letters above the bars indicate significant differences (*p* < 0.05) among the different N treatments. A,D: above-ear leaves; B,E: ear leaves; C,F: below-ear leaves. N1: 100% N applied as basal fertilizer, N2: 70% N applied as basal fertilizer and 30% N applied at the jointing stage, N3: 50% N applied as basal fertilizer and 50% N applied at the jointing stage, N4: 30% N applied as basal fertilizer, 50% N applied at the jointing stage and 20% N applied at the big flare stage.

**Fig 5:**
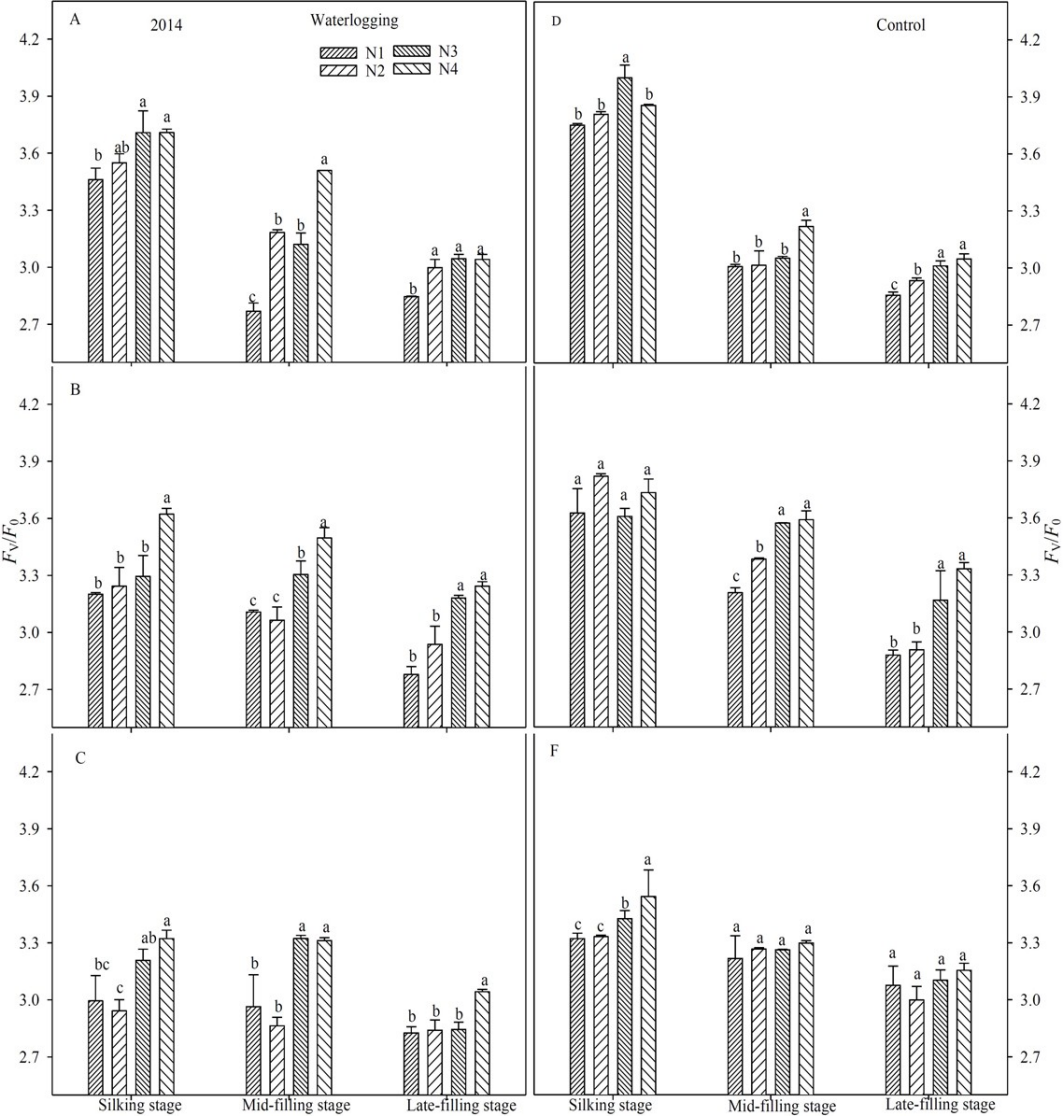
Potential efficiency of PSII (*F*_v_/*F*_0_) in different leaf layers under different N treatments (2014). The error bars represent the standard errors of the mean. Different letters above the bars indicate significant differences (*p* < 0.05) among the different N treatments. A,D: above-ear leaves; B,E: ear leaves; C,F: below-ear leaves. N1: 100% N applied as basal fertilizer, N2: 70% N applied as basal fertilizer and 30% N applied at the jointing stage, N3: 50% N applied as basal fertilizer and 50% N applied at the jointing stage, N4: 30% N applied as basal fertilizer, 50% N applied at the jointing stage and 20% N applied at the big flare stage.

**Fig 6:**
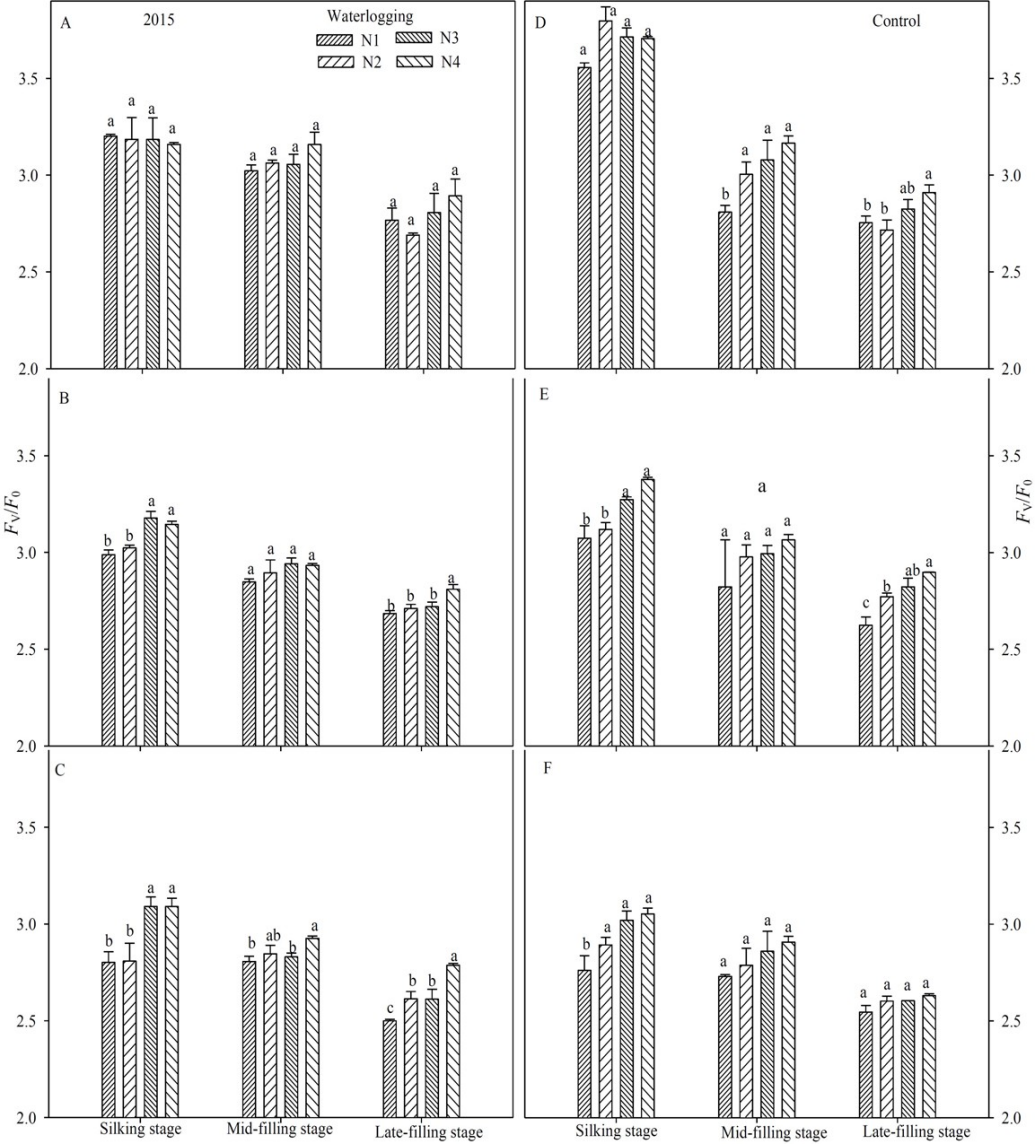
Potential efficiency of PSII (*F*_v_/*F*_0_) in different leaf layers under different N treatments (2015). The error bars represent the standard errors of the mean. Different letters above the bars indicate significant differences (*p* < 0.05) among the different N treatments. A,D: above-ear leaves; B,E: ear leaves; C,F: below-ear leaves. N1: 100% N applied as basal fertilizer, N2: 70% N applied as basal fertilizer and 30% N applied at the jointing stage, N3: 50% N applied as basal fertilizer and 50% N applied at the jointing stage, N4: 30% N applied as basal fertilizer, 50% N applied at the jointing stage and 20% N applied at the big flare stage.

**Fig 7:**
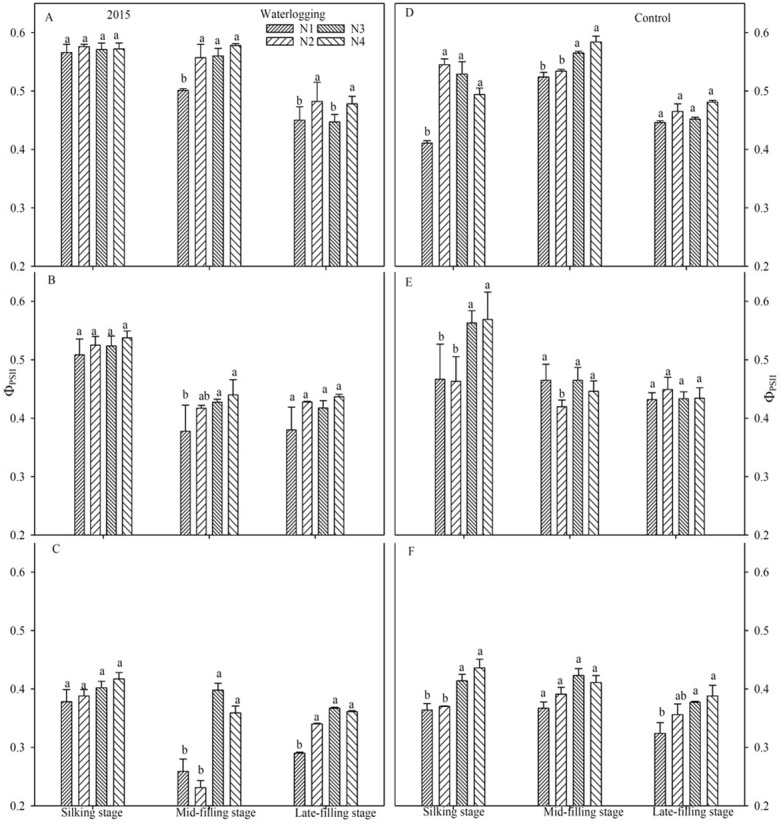
Actual quantum yield of PSII (𝚽_PSII_) in leaf layers under different N treatments (2015). The error bars represent the standard errors of the mean. Different letters above the bars indicate significant differences (*p* < 0.05) among the different N treatments. A,D: above-ear leaves; B,E: ear leaves; C,F: below-ear leaves. N1: 100% N applied as basal fertilizer, N2: 70% N applied as basal fertilizer and 30% N applied at the jointing stage, N3: 50% N applied as basal fertilizer and 50% N applied at the jointing stage, N4: 30% N applied as basal fertilizer, 50% N applied at the jointing stage and 20% N applied at the big flare stage.

Waterlogging resulted in a significant reduction of the maximal efficiency of PSII photochemistry (*F*_v_/*F*_m_) (Figs 3 and 4), the potential efficiency of PSII photochemistry (*F*_v_/*F*_o_) (Figs 5 and 6), the actual quantum yield of PSII (Φ_PSII_) (Fig 7) and the photochemical quenching of chlorophyll fluorescence (qP) (Table 4) at the silking and grain-filling stages, compared to the control. At the silking stage, the maximum values of *F*_v_/*F*_m_, *F*_v_/*F*_o_, Φ_PSII_ and qP were measured in the above-ear layer leaves, followed by the ear layer and the below-ear layer leaves. At the grain filling stage, *F*_v_/*F*_m_ and *F*_v_/*F*_o_ were ranked as ear leaf layer > above-ear layer > below-ear layer. During the grain-filling process, the *F*_v_/*F*_m_ and *F*_v_/*F*_o_ of below-ear leaves decreased the most. However, a shift in N fertilizer from a basal application to topdressing at the big flare stage alleviated waterlogging damage to *F*_v_/*F*_m_ and *F*_v_/*F*_o_. *F*_v_/*F*_m_ and *F*_v_/*F*_o_ in the N4 treatment was notably higher than in the other N treatments, with increases of 1.9~2.8% and 7.6~11.7% in the below-ear layer, 2.8~3.9% and 1.1~16.7% in the ear layer, and 1.3~5.9% and 7.1~26.8% in the above-ear layer in 2014. The values increased by 1.1~3.0% and 9.5~12.1%, 0.8~1.3% and 3.5~5.3%, and 0.9~1.2% and 5.0~8.6% for the N4 treatment, compared to the corresponding leaf layers of the N1 treatment under waterlogging in 2015.

**Table 4:** Effects of different N treatments on the photochemical quenching of variable chlorophyll fluorescence (qP) (2015).

Treatment		Above-ear leaves		Ear leaves		Below-ear leaves	
		Waterlogging	Control	Waterlogging	Control	Waterlogging	Control
Silking stage	N1	0.772±0.010 a	0.757±0.045 c	0.763±0.018 a	0.816±0.034 a	0.632±0.032 a	0.639±0.023 a
	N2	0.777±0.015 a	0.936±0.032 a	0.767±0.023 a	0.829±0.029 a	0.544±0.039 b	0.661±0.034 a
	N3	0.772±0.013 a	0.853±0.034 b	0.768±0.040a	0.834±0.045 a	0.617±0.017 a	0.665±0.026 a
	N4	0.764±0.037a	0.856±0.018 b	0.774±0.034 a	0.841±0.038 a	0.634±0.021 a	0.654±0.037 a
Mid- filling stage	N1	0.829±0.019 b	0.891±0.023 ab	0.744±0.061 b	0.941±0.041 a	0.564±0.023 b	0.777±0.023 b
	N2	0.906±0.028a	0.865±0.034 b	0.838±0.034 a	0.868±0.039 b	0.582±0.034 b	0.762±0.037 b
	N3	0.877±0.023ab	0.854±0.026 b	0.840±0.029 a	0.930±0.038 a	0.798±0.029 a	0.828±0.021 a
	N4	0.942±0.037 a	0.952±0.028 a	0.884±0.032 a	0.923±0.040 a	0.739±0.024 a	0.819±0.019 a
Late- filling stage	N1	0.860±0.023 a	0.867±0.012 a	0.768±0.029 b	0.876±0.046 a	0.613±0.018 b	0.716±0.021 b
	N2	0.826±0.029ab	0.891±0.019 a	0.820±0.023 a	0.855±0.041a	0.714±0.026 a	0.746±0.045 ab
	N3	0.834±0.039ab	0.856±0.034 a	0.822±0.032 a	0.871±0.060 a	0.715±0.034 a	0.768±0.026 a
	N4	0.814± 0.012b	0.878±0.026 a	0.822±0.025 a	0.889±0.039 a	0.723±0.023 a	0.771±0.034 a

Values followed by different letters are significantly different (*p* < 0.05) among different N treatments. N1: 100% N applied as basal fertilizer, N2: 70% N applied as basal fertilizer and 30% N applied at the jointing stage, N3: 50% N applied as basal fertilizer and 50% N applied at the jointing stage, N4: 30% N applied as basal fertilizer, 50% N applied at the jointing stage and 20% N applied at the big flare stage.

A shift in N fertilizer from a basal application to topdressing at the big flare stage had no effect on Φ_PSII_ and qP at the silking stage. However, at the mid- and late-filling stages, the highest Φ_PSII_ and qP were measured in the N4 treatment. Waterlogging decreased Φ_PSII_ in N1, N2 and N3 by 1.5~6.6% in the below-ear layer, 5.4~14.2% in the ear layer, and by 1.1~13.3% in the above-ear layer leaves, compared to the N4 treatment. The values of qP were reduced by 0.3~24.2% in the below-ear layer and 1.4~15.2% in the ear layer leaves of the N1 treatment, compared to the N4 under waterlogging. Additionally, the values of qP were decreased by 2.3~7.1% in the below-ear layer, 1.5~3.0% in the ear layer and 1.3~11.6% in the above-ear layer leaves of the N1 treatment, compared to the N4 of the control.

### Relationship between N concentration and chlorophyll fluorescence parameters

The N concentrations in the different leaf layers were significantly and positively linearly correlated with *F*_v_/*F*_m_ (*y* = 0.0447*x* + 0.6659; R^2^ = 0.7196**, *p* < 0.01) and *F*_v_/*F*_o_ (*y* = 0.5281*x* + 1.8809; R^2^ = 0.4467**, *p* < 0.01). However, there was no significant correlation between the N concentration of the different leaf layers and Φ_PSII_ or qP.

### Grain filling characteristics

**Fig 8:**
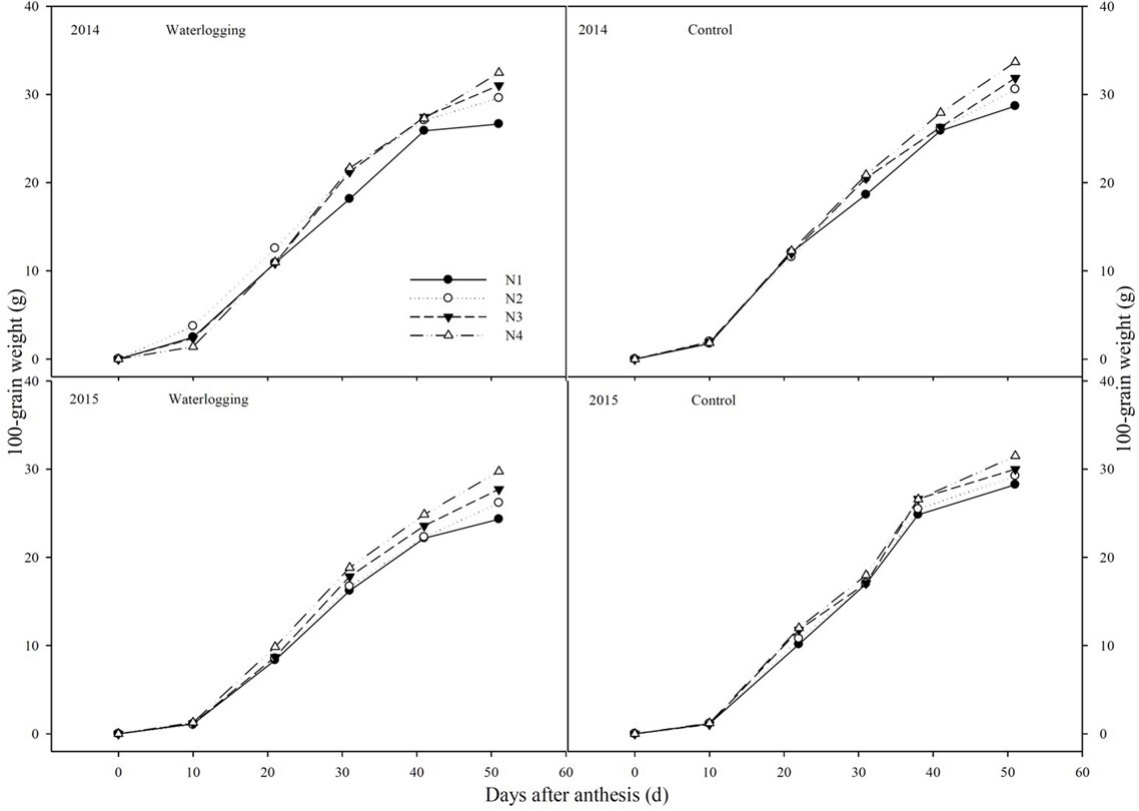
Grain-filling in different N treatments during post-anthesis stage. N1: 100% N applied as basal fertilizer, N2: 70% N applied as basal fertilizer and 30% N applied at the jointing stage, N3: 50% N applied as basal fertilizer and 50% N applied at the jointing stage, N4: 30% N applied as basal fertilizer, 50% N applied at the jointing stage and 20% N applied at the big flare stag.

Waterlogging significantly decreased the final grain weight (K) by 9.8~19.0% in 2014 and 5.5~13.9% in 2015, respectively, compared to the corresponding control (Fig 8). The grain-filling process was analyzed using Richard’s equation. According to the equation, waterlogging had adverse influences on the duration of grain filling (T) by 2.6~5.9% (Table 5). However, a shift in N from a basal application to topdressing at the big flare stage effectively alleviated the reduction of K by 5.3 and 22.3% under waterlogging and increased it by 18.0 and 12.6% for the control, compared to N1 in 2014 and 2015, respectively. Additionally, a shift in N from a basal application to topdressing at the big flare stage improved T, V_a_ and V_m_, by 0.6~7.9%, 4.4~13.0% and 2.1~20.6%, respectively, compared to other N treatments. The results indicated that a shift in N from a basal application to topdressing could alleviate the adverse effects of waterlogging on the grain filling characteristics.

**Table 5:** Effects of different nitrogen treatments on grain filling characteristics parameters under waterlogging in the seedling stage.

	2014											2015										
N treatment	*K*	*T*_m_	*V*_m_	*T*	*V*_a_	*T*_1_	*T*_2_	*T*_3_	*V*_1_	*V*_2_	*V*_3_	*K*	*T*_m_	*V*_m_	*T*	*V*_a_	*T*_1_	*T*_2_	*T*_3_	*V*_1_	*V*_2_	*V*_3_
Waterlogging																						
** **N1	27.70	25.17	0.97	57.95	0.48	15.77	18.79	23.39	0.37	0.85	0.49	24.71	26.31	0.93	56.73	0.44	17.60	17.44	21.70	0.30	0.82	0.47
** **N2	29.97	23.98	1.03	57.51	0.52	14.37	19.22	23.92	0.44	0.90	0.52	26.46	27.22	0.93	59.84	0.44	17.86	18.70	23.28	0.31	0.82	0.47
** **N3	31.27	25.84	1.13	57.63	0.54	16.73	18.22	22.68	0.40	0.99	0.57	27.94	27.24	1.00	59.30	0.47	18.05	18.38	22.87	0.33	0.88	0.50
** **N4	32.22	26.33	1.17	57.96	0.56	17.27	18.13	22.56	0.39	1.03	0.59	30.11	27.37	1.02	61.21	0.49	17.67	19.40	24.14	0.36	0.90	0.51
Control																						
** **N1	29.24	25.53	0.98	59.81	0.49	15.70	19.65	24.45	0.39	0.86	0.49	29.33	27.61	1.07	59.05	0.50	18.59	18.02	22.43	0.33	0.94	0.54
** **N2	30.64	25.76	1.05	59.28	0.52	16.16	19.21	23.91	0.40	0.92	0.53	30.53	27.89	1.07	60.57	0.50	18.52	18.73	23.32	0.35	0.94	0.54
** **N3	32.13	26.55	1.03	62.40	0.51	16.27	20.55	25.57	0.42	0.90	0.52	31.46	27.76	1.09	61.00	0.52	18.23	19.06	23.72	0.36	0.95	0.55
** **N4	34.49	27.42	1.08	64.13	0.54	16.89	21.05	26.19	0.43	0.95	0.54	33.02	28.19	1.10	62.83	0.53	18.26	19.85	24.71	0.38	0.96	0.55

K: the maximum potential grain weight; T_m_: the time reaching the maximum grain filling rate; V_m_: the maximum grain filling rate; T: duration of grain filling; V_a_: the average grain filling rate; T_1_: duration of the early grain filling period; T_2_: duration of the middle grain filling period; T_3_: duration of the late grain filling period; V_1_: filling rate of the early grain fiilling period; V_2_: filling rate of the middle grain filling period; V_3_: filling rate of the late grain filling period. N1: 100% N applied as basal fertilizer, N2: 70% N applied as basal fertilizer and 30% N applied at the jointing stage, N3: 50% N applied as basal fertilizer and 50% N applied at the jointing stage, N4: 30% N applied as basal fertilizer, 50% N applied at the jointing stage and 20% N applied at the big flare stage.

### Grain yield

Waterlogging significantly decreased the grain yield of summer maize by 7.0% in 2014 and 18.5% in 2015, compared to the control. However, a shift in N from a basal application to topdressing effectively alleviated the reduction of grain yield caused by waterlogging, and there was no interaction effect between waterlogging and N regimes. The grain yield of the N4 treatment increased by 27.0, 23.4, and 9.9% in 2014 and 25.8, 21.8, and 17.8% in 2015, compared to the N1, N2 and N3 treatments under waterlogging. The values were increased by 17.0, 12.8, and 9.6% in 2014 and 28.8, 27.9, and 21.6% in 2015, respectively, compared to the N1, N2 and N3 treatments of the control, respectively (Table 6).

**Table 6:** Effects of different nitrogen regimes on the grain yield of summer maize subjected to waterlogging at the seedling stage.

Treatment	Yield (kg hm^-2^)	1000-kernel weight (g)	Length per ear (cm)	Kernel per ear
2014 Waterlogging				
N1	5441.1±35 b	279.7±0.4 b	14.4 a	411.4 b
N2	5602.7±210 b	275.7±0.2 c	14.6 a	417.6 b
N3	6287.8±419 ab	279.6±1.2 b	14.7 a	426.3 ab
N4	6912.1±152 a	286.7±0.3 a	14.9 a	460.0 a
2014 Control				
N1	6088.4±270 b	275.3±0.6 d	15.0 a	463.5 b
N2	6316.3±167 b	280.5±0.7 c	15.4 a	482.9 a
N3	6502.8±171 ab	289.9±0.5 b	15.1 a	457.8 b
N4	7125.4±323 a	293.5±0.4 a	15.4 a	480.5a
2015 Waterlogging				
N1	4846.6±125 b	272.2±0.7 d	14.6 a	401.4 b
N2	4881.1±238 b	280.3±0.2 c	14.5 a	411.8 b
N3	5132.3±322 b	285.5±0.3 b	14.8 a	410.0 b
N4	6243.4±328 a	293.4±0.3 a	14.6 a	450.1 a
2015 Control				
N1	5945.7±359 b	287.4±0.2 d	14.8 a	430.9 b
N2	5988.0±312 b	282.7±0.1 c	14.7 a	430.8 b
N3	6296.7±345 b	291.0±0.3 b	14.9 a	435.5 b
N4	7659.3±346 a	298.0±0.4 a	14.8 a	460.1 a

Values followed by different letters are significantly different (*p* < 0.05) among different N treatments. N1: 100% N applied as basal fertilizer, N2: 70% N applied as basal fertilizer and 30% N applied at the jointing stage, N3: 50% N applied as basal fertilizer and 50% N applied at the jointing stage, N4: 30% N applied as basal fertilizer, 50% N applied at the jointing stage and 20% N applied at the big flare stage.

## Discussion

### Adverse effects of waterlogging on gLA and leaf photosynthetic capacity

Waterlogging affects crop growth and development [5, 7, 22], and decreases maize gLA [6]. In this study, waterlogging significantly decreased the gLA of maize, which was in agreement with previous studies [6, 20]. The reduction of gLA under waterlogging may related to the decline in ZR, IAA and GA contents, and the increase in leaf ABA contents, resulting in an acceleration of leaf senescence [12, 13]. Interestingly, waterlogging significantly decreased gLA in the below-ear and above-ear layer leaves. The reduction of gLA in the below-ear layer may due to the decline in the absorption ability of root, which inhibits N absorption and transport [23], resulting in a deficiency of N in below-ear layer leaves and an acceleration of leaf senescence.

The gLAI is an important indicator of the canopy architecture at different growth stages, and a longer gLA duration and photosynthetic capacity during the post-silking period is one of several traits associated with an improvement in maize yield [39]. Our study showed that waterlogging caused a significant reduction in P_N_ and g_s_ accompanied by a significant increase in c_i_ in the ear leaves, indicating that non-stomatal closure was the main reason for the decrease in P_N_, which differed from previous report [20]. Moreover, waterlogging had negative effect on *F*_v_/*F*_m_, *F*_v_/*F*_o_, Φ_PSII_ and qP, especially for the below-ear layer leaf values. Photosynthates of the below-ear layer leaves mainly supply root growth. Under waterlogging conditions, diffusion of gases through soil pores is inhibited, resulting in enhanced of anaerobic respiration and accumulation of harmful substances in soil, and deterioration of rhizosphere environments [14, 21,40]. Root development is constrained, which leads to abnormal growth of shoot parts [7, 20], especially the below-ear layer leaves. Furthermore, the decline of gLA may cause an increase in light transmittance and excessive light leakage losses, consequently decreasing irradiation energy utilization efficiency and P_N_ [4, 41]. Optimum canopy function is a key factor in achieving a higher yield [42, 43]. The grain number and 1000-grain weight are established in the grain filling stage [44]. It was clear that waterlogging reduced the photosynthetic capacity, by decreasing the leaf N concentration, gLA and P_N_, indicating a reduction in “source” characteristics. In addition, waterlogging decreased the grain-filling duration (T). The “source” characteristics were constrained, resulting in a reduction of “sink” (i.e., grain yield decreases) characteristics for waterlogged summer maize.

### The compensating effect of the N regime on the development of maize

Nitrogen (N) is essential in the synthesis of the photosynthetic apparatus, which is closely related to the gLA and P_N_ [36, 41, 45, 46]. However, the compensating effects of N on gLA, N concentration and photosynthetic capacity in different leaf layers of summer maize subjected to waterlogging at the seedling stage remain unclear. Our results indicated that a shift in N from a basal application to topdressing at the big flare stage increased N concentration and gLA in the leaves, especially in the ear layer and above-ear layer leaves, which alleviated the decline in gLA induced by waterlogging, thus increasing the gLA of the ear and above-ear layers and compensating for the gLA decrease in the below-ear layer leaves. The compensating effect of N was significant and ultimately resulted in an increase in gLA compared to 100% basal fertilizer application. N element can increase cytokinin synthesis in the roots and subsequent transport to the leaves [47], and enhance the new leaf area expansion, eventually increasing gLA, dry matter accumulation and transport.

N also enhances root and shoot development through increased maize root exudation and total abundance of soil bacteria [48]. Low leaf N concentrations have a negative effect on the P_N_ [36, 49]. N deficiency can decrease the activity of Rubisco and PEPC enzymes, and the degradation of Rubisco and PEPC can decrease the photosynthetic capacity during the leaf senescence process [36]. Additionally, supplying N increases the chlorophyll content and P_N_ of plants [36, 41]. Our results showed that with the improvement in the gLA and N concentration, the photosynthetic capacity was improved at the ear layer leaves in the later filling stage, and the chlorophyll fluorescence parameters PSII was improved at the ear layer and above-ear layer leaves, indicating that the supplied N could increase the effective quantum yield of photochemical energy conservation in PSII, which is in agreement with previous studies [4, 36, 50]. Photosynthates of above-ear layer leaves supply tassel and grains, while those of the ear layer mainly supply grain filling. A shift in N from basal application to topdressing at the big flare stage was conducive to alleviating the decrease in the grain yield, by maintaining a higher gLA, P_N_, *F*_v_/*F*_m_, and N concentration in the ear layer and above-ear layer leaves, and a higher grain filling rate during the grain-filling period, ultimately alleviating waterlogging damages. Simultaneously, our results indicated that waterlogging inhibited the gLA and photosynthetic capacity, which was improved, but not reversed, by the N regimes. This phenomenon may due to the deterioration of morphological characteristics and the decline of the absorption area of root under waterlogging [4], which leads to abnormal development of the canopy. Improvement of the compensating effects to reach an optimum status still requires future research via the improvement of root and the interaction of root and shoot by N regimes. Consequently, changing the normal fertilization method (100% basal fertilizer) to a combination of basal and topdressing application is recommended to improve maize growth in areas subjected to waterlogging during the seedling stage.

## Conclusion

Waterlogging at the seedling stage had significantly adverse effects on gLA, N concentration, and photosynthetic capacity, especially in the below-ear layer leaves. A shift in N from basal application to topdressing at the big flare stage could compensate for the adverse effects of waterlogging, by increasing the gLA, N concentration, and photosynthetic capacity in the ear and above-ear layer leaves. This greater leaf photosynthesis increased grain yield primarily via improved grain-filling rate. Overall, this study indicated that shifting the N application to the big flare stage was able to compensate for the adverse effects, but could not reverse the adverse effects caused by waterlogging at the seedling stage.

## Supporting information

S1 File This excel file includes green leaf area in different leaf layers at the silking stage. The effects of different nitrogen regimes on the green leaf area of different leaf layers at the silking stage are based on this dataset.
(XLSX)

S2 File This excel file includes nitrogen concentration of different leaf layers at the silking and maturity stages. The effects of different nitrogen regimes on nitrogen concentration of different leaf layers are based on this dataset.
(XLSX)

S3 File This excel file includes photosynthetic rate at the silking stage. The effects of different nitrogen regimes on the photosynthetic capacity at the silking stage are based on this dataset.
(XLSX)

S4 File This excel file includes chlorophyll fluorescence parameters of different leaf layers at different growth stages. The effects of different nitrogen regimes on the chlorophyll fluorescence parameters of different leaf layers at different growth stages are based on this dataset.
(XLSX)

S5 File This excel file includes grain weight during grain filling stage. The effects of different nitrogen regimes on grain weight during grain filling stage are based on this dataset.
(XLSX)

S6 File This excel file includes grain yield. The effects of different nitrogen regimes on the grain yield of summer maize subjected to waterlogging at the seedling stage.
(XLSX)
